# A Fatal Case of Cardiac Sarcoidosis Presenting as Refractory Ascites

**DOI:** 10.7759/cureus.85718

**Published:** 2025-06-10

**Authors:** Ernestine Faye S Tan, Sakar B Gharti, Marie Schmidt, Nabin K C, Danilo Enriquez

**Affiliations:** 1 Internal Medicine, Interfaith Medical Center, New York, USA; 2 Pulmonary and Critical Care Medicine, Interfaith Medical Center, New York, USA

**Keywords:** arrhythmia, ascites, cardiac sarcoidosis, cardiology, heart block, heart failure, heart failure with reduced ejection fraction, liver sarcoidosis, peritoneal sarcoidosis, sarcoidosis

## Abstract

Sarcoidosis is a multisystem granulomatous disease with variable clinical presentations, most commonly affecting the lungs. Cardiac sarcoidosis (CS), though rare, is a serious complication that can lead to restrictive cardiomyopathy and carries a poor prognosis. We report the case of a 44-year-old male patient who presented with worsening ascites, fatigue, orthopnea, and peripheral edema. Initial workup revealed transudative ascites and new-onset severe heart failure, with echocardiography showing an ejection fraction of 15%-20%, bi-atrial enlargement, left ventricular hypertrophy, and grade 3 diastolic dysfunction. Cardiac catheterization confirmed non-ischemic cardiomyopathy. Imaging showed pulmonary and mediastinal lymphadenopathy and ground-glass opacities. Due to the patient's instability, a biopsy was deferred, and a myocardial fluorodeoxyglucose-18 positron emission tomography (FDG-PET) scan was performed, demonstrating diffuse hypermetabolic activity consistent with active CS. Despite immunosuppressive therapy with prednisone, methotrexate, hydroxychloroquine, and folate, the patient deteriorated and succumbed to fatal arrhythmias and advanced conduction abnormalities. This case highlights the diagnostic utility of FDG-PET in unstable patients and underscores the importance of considering CS in patients with unexplained heart failure and transudative ascites. Early recognition and consideration for cardiac transplant are critical in improving outcomes.

## Introduction

Sarcoidosis is a multisystem, inflammatory, granulomatous disease characterized by the accumulation of T-lymphocytes and mononuclear phagocytes in solid organs, resulting in the disease’s characteristic non-caseating granulomas [1,2]. To date, the origin and pathophysiology of sarcoidosis remain inadequately understood. The lung is the most common organ involved, with up to 90% of the cases presenting with respiratory symptoms, but occasionally, sarcoidosis can present only with extrapulmonary involvement, such as the liver and the peritoneal cavity [1-3]. Other organs may also be involved, including the heart, the lymph nodes (LNs), the skin, the eyes, the kidneys, the musculoskeletal system, and parts of the central nervous system [4].

The prevalence of cardiac sarcoidosis (CS) in the United States and Europe is approximately 10-40 per 100,000 members of the population, more common in the African American population (35.5 per 100,000) than in the White or Caucasian population (11 per 100,000) [1,4].

CS is oftentimes an underrecognized disease entity. Approximately 40%-50% of patients diagnosed with CS postmortem were merely incidental findings and had not been diagnosed in their lifetime [2]. It is also estimated that 20%-30% of pulmonary or systemic sarcoidosis patients with cardiac involvement have clinically silent cardiac disease [5]. However, for those who have cardiac manifestations, the prognosis is often poor and the symptoms fatal. Cardiac involvement accounts for as much as 25% of all deaths from sarcoidosis in the United States and up to approximately 85% in the Japanese population with sarcoidosis [4].

The exact pathophysiology of CS remains incompletely understood. The diagnosis of CS relies on both histologic and clinical presentations; however, due to the non-specific myriad of symptoms that may affect any organ in the body, there is a lack of clinical consensus guidelines on the approach to diagnosis, resulting in often delayed detection and treatment. It has been postulated that the clinical presentation of CS depends on the location, extent, and activity of the myocardial infiltration [3]. In a study by Cheng and associates, they mentioned that involvement of the interventricular septum is more likely to present with heart block, whereas extensive myocardial fibrosis is more likely to develop heart failure and ventricular dysfunction [3].

There is no reliable biomarker to predict the presence or severity of the disease. Despite the limited data on CS, the most important predictors of prognosis are the extent of left ventricular (LV) dysfunction, the extent of right ventricular (RV) dysfunction, and the extent of myocardial late gadolinium enhancement [3,6]. As patients start to show cardiac manifestations, the risk of malignant arrhythmias and sudden cardiac death dramatically increases, emphasizing the importance of early diagnosis and a high index of suspicion [7]. We are presenting a case of CS with an unusual presentation of refractory ascites.

## Case presentation

This is a case of a 44-year-old male patient who presented with worsening and recurrent ascites. The patient has a known case of hypertension, diabetes mellitus type 2, mild intermittent bronchial asthma, end-stage renal disease (ESRD), cannabinoid use, morbid obesity, and chronic combined systolic and diastolic heart failure. He presented to the emergency department with abdominal pain, abdominal girth enlargement, and fever.

History started four years prior to the current presentation when he was brought to the emergency room for evaluation of shortness of breath and was found to have new-onset congestive heart failure (CHF), for which he was treated with diuretics. At this time, an echocardiogram revealed LV ejection fraction (LVEF) of 10%-15% and severe concentric LV hypertrophy, bi-atrial dilatation, reduced RV systolic function, and grade 3 severe LV diastolic dysfunction. Pulmonary artery systolic pressure was elevated at 46.36 mmHg. Cardiac catheterization revealed non-ischemic heart failure, and the patient was treated conservatively with diuretics (spironolactone and metolazone), dapagliflozin, and sacubitril-valsartan.

**Figure 1:**
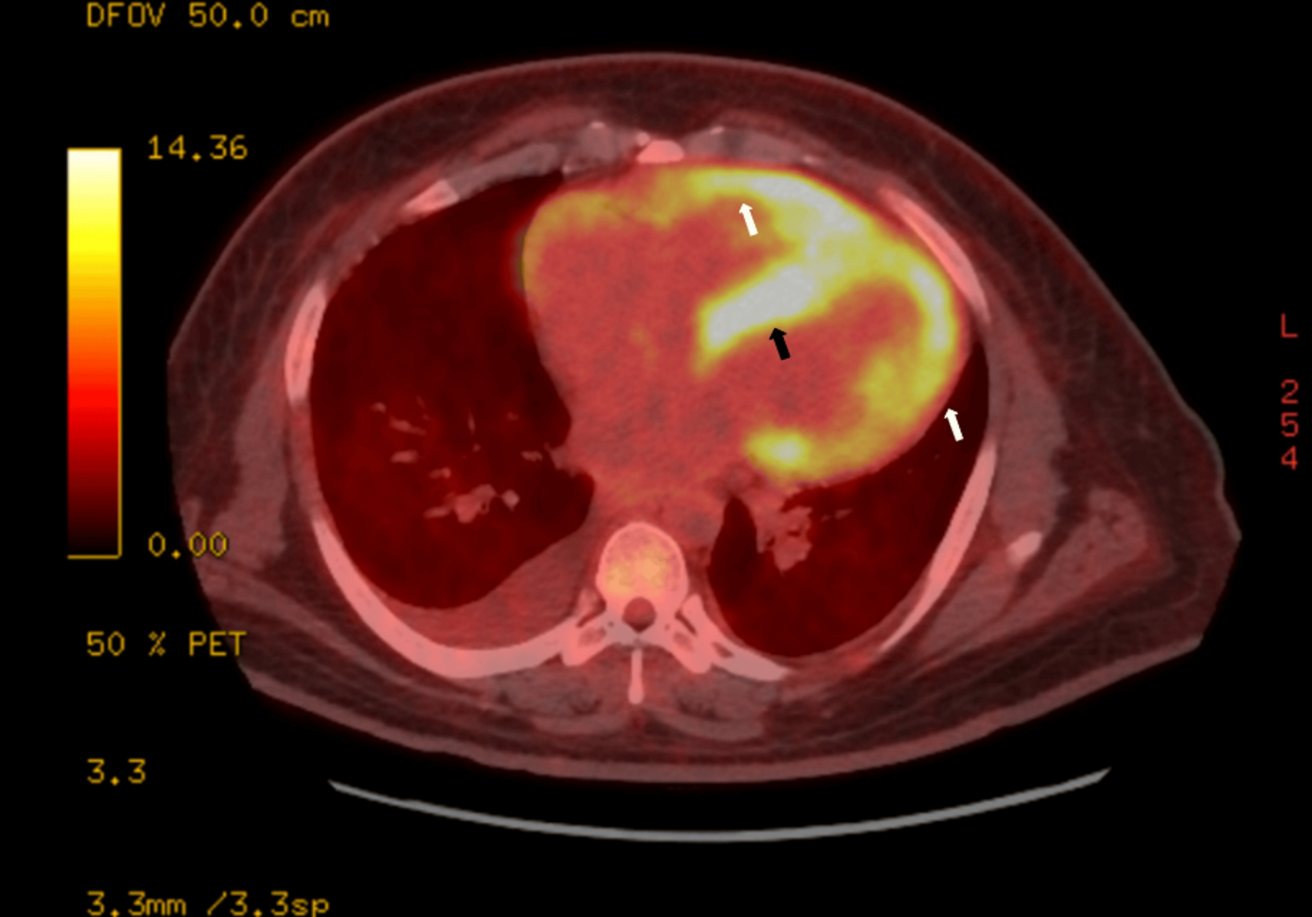
FDG-PET showing diffuse, increased hypermetabolic activity (yellow enhancement) on both ventricles (white arrows), greatest in the interventricular septum (black arrow) FDG-PET: fluorodeoxyglucose-18 positron emission tomography

The patient was admitted several times for acute decompensation of CHF, and he subsequently developed lower extremity edema and abdominal distension, along with orthopnea and paroxysmal nocturnal dyspnea. However, he denied cough, fever, chills, or exposure to sick contacts. On current admission, his vitals were stable; he was afebrile at 37.2°C, with a blood pressure of 110/78 mmHg, a heart rate of 99 beats per minute, and a respiratory rate of 20 beats per minute, despite increased work of breathing. A computed tomography (CT) of the chest was done, which revealed bilateral pleural effusions, mild to moderate mediastinal and pulmonary hilar lymphadenopathies, and ground-glass opacities, raising concern for sarcoidosis. Endobronchial ultrasound with biopsy (EBUS) was planned; however, due to poor functional capacity and frequent heart failure exacerbations, the procedure was delayed for medical optimization. Instead, a fluorodeoxyglucose-18 positron emission tomography (FDG-PET) scan of the myocardium was done, which revealed diffuse, increased hypermetabolic activity in both ventricles, greatest in the interventricular septum, as well as mild increased hypermetabolic activity in the walls of the right atrium, consistent with active CS (Figure 1). Interestingly, the enlarged mediastinal LNs found on chest CT were not significantly hypermetabolic.

The patient was started on prednisone 40 mg daily and later on added methotrexate, folate, and hydroxychloroquine, which slightly improved the symptoms; however, he continued to have decreased functional capacity with no improvement in the LVEF, and he was prescribed a wearable cardioverter defibrillator.

He clinically deteriorated and ultimately developed atrial fibrillation, with episodes of atrial flutter, for which he had to be cardioverted and started on apixaban. He had a brief episode of cardiac arrest with successful resuscitation. He developed episodes of atrioventricular (AV) block 3:1 and was referred to an electrophysiologist for ablation and an automated implantable cardioverter defibrillator (AICD).

His CHF continued to worsen; he ultimately developed hepatic cirrhosis from chronic passive liver congestion versus liver sarcoid (LS) and ESRD, for which he underwent hemodialysis. Despite treatment, he had worsening ascites, abdominal distention, fever, nausea, vomiting, and inability to tolerate food. He eventually developed hepatic encephalopathy, for which he was started on lactulose. He underwent an abdominal tap, and a pigtail catheter was placed, which was used for therapeutic paracentesis at least three times a week. Due to his septic presentation, the patient was treated with cefepime and vancomycin for consideration of spontaneous bacterial peritonitis. Ascites were initially transudative in nature; however, subsequent blood cultures during the hospital course grew vancomycin-resistant *Enterococcus faecalis* (VRE), and peritoneal fluid grew *Candida auris*, likely secondary to infection from the indwelling pigtail catheter. The patient was started on daptomycin, ceftaroline, and fluconazole, and a repeat echocardiogram was done to rule out infective endocarditis. Echocardiogram revealed an LVEF of 15%-20%, grade III diastolic dysfunction (restrictive), global hypokinesis of the LV, LV hypertrophy, bi-atrial dilatation, and small pulmonic valve vegetation, which was 1.6 cm x 1 cm in size. The RV systolic pressure (RVSP) was 88 mmHg. The patient was planned for a transesophageal echocardiogram but continued to be unstable, with hypotension, desaturation, tachycardia, and lethargy. Ultimately, he was suddenly noted to have pulseless electrical activity, and resuscitation was done without return of spontaneous circulation.

## Discussion

To date, endomyocardial biopsy (EMB) remains to be the gold standard for diagnosis of CS [1,8]. Unfortunately, this procedure is invasive and the sensitivity is low, with a yield of only 25%-36% due to the patchy involvement of the myocardium in most cases [1,8]. Definitive diagnosis can be demonstrated by the presence of non-caseating granulomas, which are the histopathologic hallmarks of the disease [1,8,9]. Should a biopsy be difficult to perform, sufficient diagnostic likelihood can be achieved by combining extracardiac histology of sarcoidosis with clinical manifestations and findings on cardiac imaging [9].

Echocardiography in patients with CS may reveal regional wall motion abnormalities, systolic dysfunction, and LV dilatation, although the sensitivity is low [1,3,4,9]. Oftentimes, patients have normal echocardiograms during the initial stages [3]. In our patient who presented with new-onset, unexplained, non-ischemic heart failure, the echocardiogram revealed the first clue of sarcoidosis when the systolic function was found to be severely depressed at 10%-15% and grade 3 diastolic function. Chest radiography also revealed hilar and mediastinal lymphadenopathies, which led to a suspicion of sarcoidosis. Our patient could not undergo EMB; he was too unstable. Cardiac magnetic resonance imaging (MRI) (CMRI) was not available at the time, and the patient eventually underwent FDG-PET, which confirmed the likelihood of the disease. It has been postulated that the clinical presentation of CS depends on the location, extent, and activity of the myocardial infiltration [3]. In a study by Cheng and associates, they mentioned that involvement of the interventricular septum is more likely to present with heart block, whereas extensive myocardial fibrosis is more likely to develop heart failure and ventricular dysfunction [3].

FDG-PET is a functional study that detects inflammatory cells with a very high metabolic rate; a positive test has an estimated sensitivity of 89% and specificity of 78% [10]. On the other hand, CMRI detects structural changes like wall thinning, aneurysms, and chamber dilation [1,3,4,9]. Late gadolinium enhancement, a poor prognostic indicator, is a critical diagnostic tool that usually indicates myocardial fibrosis and edema [11,12]. The sensitivity of CMRI for detecting CS is 75%-100%, and the specificity is 76%-100% [13].

It has been postulated that the clinical presentation of CS depends on the location, extent, and activity of the myocardial infiltration [3]. In a study by Cheng and associates, they mentioned that involvement of the interventricular septum is more likely to present with heart block, whereas extensive myocardial fibrosis is more likely to develop heart failure and ventricular dysfunction [3]. The patient’s FDG-PET scan revealed increased hypermetabolic activity greater in the interventricular septum and bilateral ventricles. The interventricular septum involvement explained his AV block, while the biventricular involvement also explained the heart failure. Meanwhile, the abnormalities of the right atrium could also explain the fatal flutter-fibrillation component that required cardioversion and, eventually, ablation. While there were mildly enlarged mediastinal LNs on chest radiographs, the hypermetabolic uptake on FDG-PET, which is usually the hallmark of sarcoidosis, was only mild in our patient. A review of the literature revealed reports of CS without lung involvement. Two cases of isolated CS were published, respectively, by Ather and associates and Okada et al. [14,15]. In both cases, there were no lung involvements, and CS was confirmed by cardiac imaging [14,15].

The infiltrative nature of this disease has led to liver cirrhosis, which ultimately led to several complications, including refractory ascites. Ascites is a rare but possible manifestation of sarcoidosis and can be caused by severe LS, CHF, or peritoneal sarcoidosis when granulomas are present [16-18]. Normally, the peritoneal involvement of sarcoidosis would result in exudative ascites [19], but the transudative nature of our patient’s ascitic fluid highly suggested heart failure and liver cirrhosis as the primary culprits. This prompted a further investigation into a 44-year-old male patient with unexplained liver cirrhosis, heart failure, and refractory ascites, ultimately leading us to do CT scans and an FDG-PET.

**Figure 2:**
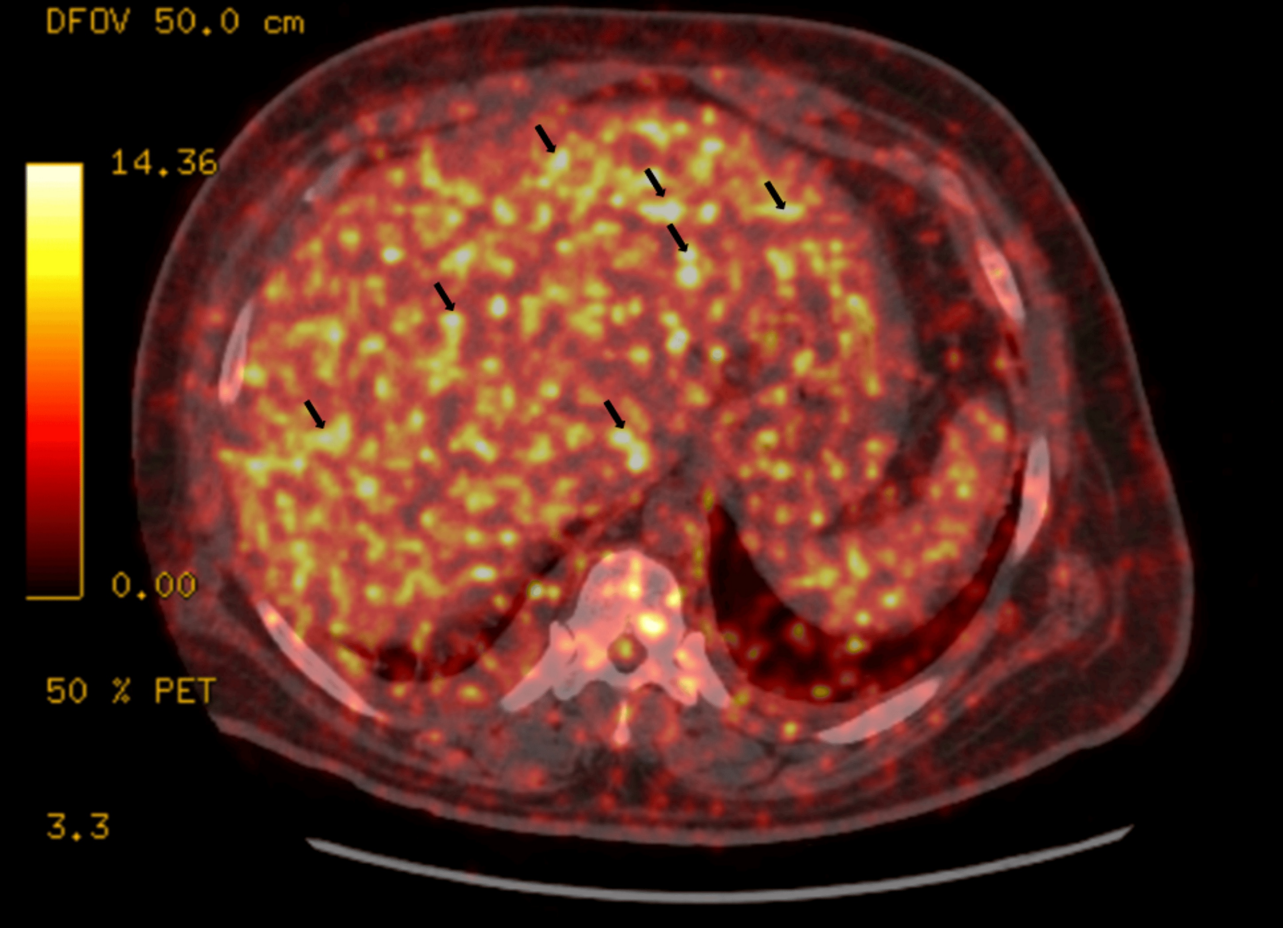
FDG-PET showing hepatomegaly, with diffuse, increased hypermetabolic activity on the liver (bright yellow enhancements, as indicated by black arrows) FDG-PET: fluorodeoxyglucose-18 positron emission tomography

The diffuse, hypermetabolic activity of the liver in FDG-PET supported LS (Figure 2). Peritoneal involvement is extremely rare in sarcoidosis and was not suspected in our case, given the transudative nature of ascitic fluid on initial presentation. Abdominal CT scan and FDG-PET did not show any abnormality in the peritoneal cavity. An extensive workup for cirrhosis was negative for autoimmune and rheumatologic etiologies: Antinuclear antibodies (Ab) (ANA) were 1:320 with a speckled pattern; anti-Jo-1, anti-double-stranded deoxyribonucleic acid (anti-dsDNA), anti-RNP (ribonucleoprotein) Ab, anti-smooth muscle Ab, anti-mitochondrial Ab, and anti-Smith Ab were all negative. Labs were significant for mild transaminitis, hyperbilirubinemia, and a significantly elevated alkaline phosphatase, which ranged between 250 and 429 IU/L (normal value: 30-130 IU/L). Magnetic resonance cholangiopancreatography (MRCP) was not done due to hemodynamic instability. Despite the initiation of dialysis, the patient continued to have significant refractory ascites. LS is rare, but in view of hepatosplenomegaly on the abdominal CT scan, increased uptake in the liver on FDG-PET, and marked elevation of alkaline phosphatase, we believed that our patient had LS. Although a biopsy would have confirmed this diagnosis, the patient was too unstable to undergo such a procedure.

Patients with systemic sarcoidosis and CS, as in our patient, have a worse prognosis than those with non-cardiac involvement [19]. Heart failure as a clinical manifestation and reduced LVEF carry an especially poor prognosis with a reported 10-year survival of 19% to 53% in the absence of cardiac transplant [20]. Immunosuppressive therapy remains the cornerstone of management in patients with CS [19,20]. In most cases, corticosteroids are the acceptable first-line agents to control disease progression [21,22]. Other agents like methotrexate and azathioprine are often used as steroid-sparing agents or as first-line treatment in patients unable to tolerate steroids. In a retrospective study by Kandolin and associates between 1988 and 2012, 110 patients with histologically confirmed CS were identified. The researchers followed their disease progression. They found that 65 patients with a reduced EF (<50%) on diagnosis showed no overall change over 12 months of steroid therapy. The study also found that the one-, five-, and 10-year transplantation-free cardiac survival was 97%, 90%, and 83%, respectively. Heart failure at presentation predicted poor outcomes (log-rank p = 0.0001) with a 10-year transplantation-free cardiac survival of only 53% [23,24]. For this reason, prednisone therapy was initiated early, despite EMB being postponed on several occasions due to poor clinical status. In a study by Velangi and colleagues of 290 patients with CS, RV systolic dysfunction was independently associated with all-cause death [20].

For heart failure patients with reduced EF, guideline-directed medical therapy (GDMT) must be initiated, which includes β-blockers, renin-angiotensin blockade including angiotensin receptor neprilysin inhibition, mineralocorticoid receptor antagonists, and sodium-glucose cotransporter-2 inhibitors [3,22]. These help manage LV dysfunction [22]. Diuretics should be used for the symptomatic management of volume overload [3,22]. GDMT may help delay the progression of cardiac involvement and improve LV dysfunction [3,22,25]. Dysrhythmias must also be managed with antiarrhythmics, as well as rate and rhythm control. Ablation is commonly reserved for those refractory to medical therapy [25]. The choice to anticoagulate in patients with atrial fibrillation or flutter, as in our patient, must be guided by the CHA2DS2-VASc score [1]. Our patient had a CHA2DS2-VASc score of 5; hence, apixaban was an essential addition to therapy. An AICD is recommended for patients with sustained ventricular arrhythmias or prior cardiac arrest or those with LVEF less than 35% despite medical therapy and immunosuppression [22]. AICD therapy is useful in patients with unexplained syncope or presyncope of likely arrhythmic etiology or inducible sustained ventricular arrhythmia [26]. However, as in most medical conditions, therapy must always be individualized as per the patient’s clinical symptoms. In our patient, AICD was placed for chronic progressive heart failure with a reduced EF of 10%-15%. Despite this, the patient developed sudden cardiac death.

For LS and ascites, the treatment is that of sarcoidosis with immunosuppression and treatment of heart failure. In ascites with portal hypertension, transjugular intrahepatic portosystemic shunt (TIPS) may be considered. In a study of 32 patients, Rosemurgy and associates suggested that TIPS may be used to treat medically intractable ascites and that it was better than a peritoneovenous shunt in terms of long-term efficacy [27]. They also found that survival rates were higher in the TIPS group, and it is a good alternative for poor transplant candidates with an expected survival of more than three months [27]. Large-scale studies, however, have yet to be done regarding this.

Young patients with progressive cardiomyopathy refractory to all therapy should undergo evaluation for cardiac transplantation [28]. A study by Jackson and colleagues in 2021 showed that patients with CS who underwent cardiac transplantation had similar post-transplant survival, odds of graft failure, hospitalization for infection, and post-transplant malignancy to those who had transplantation for non-sarcoidosis reasons [29]. This reflects that transplantation is a viable option for CS [29].

## Conclusions

CS is a rare and often overlooked condition that can mimic other heart diseases, requiring a high level of clinical suspicion for diagnosis. It should be considered in patients with new heart failure, arrhythmias, or heart block. Although EMB is the diagnostic gold standard, non-invasive imaging (like CMRI and FDG-PET) and biopsies from extracardiac sites can aid in diagnosis.

Systemic sarcoidosis can present atypically with complications like ascites and liver cirrhosis, which are harder to treat and may require palliative care or liver transplantation, though outcomes are uncertain. There is no cure, but early diagnosis and treatment with immunosuppressive therapy can improve survival and manage symptoms. Treatment may involve medications (steroids, immunomodulators, and antiarrhythmics), devices (AICDs), and, in some cases, heart transplants.

## References

[1] Hussain K, Shetty M (2025). Cardiac sarcoidosis. StatPearls [Internet].

[2] Lee SJ, Kim EH, Kim YS (2012). A case of sarcoidosis combined with massive ascites. J Rheumatol Dis.

[3] Cheng RK, Kittleson MM, Beavers CJ (2024). Diagnosis and management of cardiac sarcoidosis: a scientific statement from the American Heart Association. Circulation.

[4] Houston BA, Mukherjee M (2014). Cardiac sarcoidosis: clinical manifestations, imaging characteristics, and therapeutic approach. Clin Med Insights Cardiol.

[5] Patel MR, Cawley PJ, Heitner JF (2009). Detection of myocardial damage in patients with sarcoidosis. Circulation.

[6] Saeed M, Wagner S, Wendland MF, Derugin N, Finkbeiner WE, Higgins CB (1989). Occlusive and reperfused myocardial infarcts: differentiation with Mn-DPDP--enhanced MR imaging. Radiology.

[7] Locke AH, Gurin MI, Sabe M, Hauser TH, Zimetbaum P (2021). Arrhythmia in cardiac sarcoidosis. Cardiol Rev.

[8] Shah HH, Zehra SA, Shahrukh A (2023). Cardiac sarcoidosis: a comprehensive review of risk factors, pathogenesis, diagnosis, clinical manifestations, and treatment strategies. Front Cardiovasc Med.

[9] Birnie DH, Nery PB, Ha AC, Beanlands RS (2016). Cardiac sarcoidosis. J Am Coll Cardiol.

[10] Bois JP, Muser D, Chareonthaitawee P (2019). PET/CT evaluation of cardiac sarcoidosis. PET Clin.

[11] Stevenson A, Bray JJ, Tregidgo L (2023). Prognostic value of late gadolinium enhancement detected on cardiac magnetic resonance in cardiac sarcoidosis. JACC Cardiovasc Imaging.

[12] Hulten E, Agarwal V, Cahill M (2016). Presence of late gadolinium enhancement by cardiac magnetic resonance among patients with suspected cardiac sarcoidosis is associated with adverse cardiovascular prognosis: a systematic review and meta-analysis. Circ Cardiovasc Imaging.

[13] Blankstein R, Kramer CM, Chandrashekhar Y (2017). The challenges of diagnosing cardiac sarcoidosis. JACC Cardiovasc Imaging.

[14] Ather K, Parulkar SS, Levine D, Tran C, Atalay MK, Apostolidou E (2021). A case of isolated cardiac sarcoidosis diagnosed with multimodality cardiac imaging. CASE (Phila).

[15] Okada DR, Bravo PE, Vita T (2018). Isolated cardiac sarcoidosis: a focused review of an under-recognized entity. J Nucl Cardiol.

[16] Hackworth WA, Kimmelshue KN, Stravitz RT (2009). Peritoneal sarcoidosis: a unique cause of ascites and intractable hiccups. Gastroenterol Hepatol (N Y).

[17] Wajid M, Michaeli G, Birner Z, Sharif A, Khan S, Rahman O (2023). Ascites in sarcoidosis. Am J Respir Crit Care Med.

[18] Gorkem U, Gungor T, Bas Y, Togrul C (2015). Abdominal sarcoidosis may mimic peritoneal carcinomatosis. Case Rep Obstet Gynecol.

[19] Kim JS, Judson MA, Donnino R, Gold M, Cooper LT Jr, Prystowsky EN, Prystowsky S (2009). Cardiac sarcoidosis. Am Heart J.

[20] Velangi PS, Chen KA, Kazmirczak F (2020). Right ventricular abnormalities on cardiovascular magnetic resonance imaging in patients with sarcoidosis. JACC Cardiovasc Imaging.

[21] Giblin GT, Murphy L, Stewart GC (2021). Cardiac sarcoidosis: when and how to treat inflammation. Card Fail Rev.

[22] Gilotra N, Okada D, Sharma A, Chrispin J (2020). Management of cardiac sarcoidosis in 2020. Arrhythm Electrophysiol Rev.

[23] Kandolin R, Lehtonen J, Airaksinen J (2015). Cardiac sarcoidosis: epidemiology, characteristics, and outcome over 25 years in a nationwide study. Circulation.

[24] Sykora D, Bratcher M, Churchill R (2024). Medical therapy and clinical outcomes in cardiac sarcoidosis patients with systolic heart failure. Circ J.

[25] Birnie DH, Sauer WH, Bogun F (2014). HRS expert consensus statement on the diagnosis and management of arrhythmias associated with cardiac sarcoidosis. Heart Rhythm.

[26] Francisco Pascual J, Jordan Marchite P, Rodríguez Silva J, Rivas Gándara N (2023). Arrhythmic syncope: from diagnosis to management. World J Cardiol.

[27] Rosemurgy AS, Zervos EE, Clark WC (2004). TIPS versus peritoneovenous shunt in the treatment of medically intractable ascites: a prospective randomized trial. Ann Surg.

[28] Mantini N, Williams B Jr, Stewart J, Rubinsztain L, Kacharava A (2012). Cardiac sarcoid: a clinician's review on how to approach the patient with cardiac sarcoid. Clin Cardiol.

[29] Jackson KC, Youmans QR, Wu T (2021). Heart transplantation outcomes in cardiac sarcoidosis. J Heart Lung Transplant.

